# A Non-Synonymous Single Nucleotide Polymorphism in the *HJURP* Gene Associated with Susceptibility to Hepatocellular Carcinoma among Chinese

**DOI:** 10.1371/journal.pone.0148618

**Published:** 2016-02-10

**Authors:** Wenfeng Huang, Hongxing Zhang, Yumin Hao, Xiaobing Xu, Yun Zhai, Shaoxia Wang, Yang Li, Fuchao Ma, Yuanfeng Li, Zhifu Wang, Yang Zhang, Xiumei Zhang, Renxiang Liang, Zhongliang Wei, Ying Cui, Yongqiang Li, Xinsen Yu, Hongzan Ji, Fuchu He, Weimin Xie, Gangqiao Zhou

**Affiliations:** 1 State Key Laboratory of Proteomics, Beijing Proteome Research Center, Beijing Institute of Radiation Medicine, Beijing, China; 2 National Engineering Research Center for Protein Drugs, Beijing, China; 3 National Center for Protein Sciences Beijing, Beijing, China; 4 Affiliated Cancer Hospital of Guangxi Medical University, Nanning, Guangxi, China; 5 The Third Affiliated Hospital of Guangxi Medical University, Nanning, Guangxi, China; 6 Department of Gastroenterology and Hepatology, Jinling Hospital, Clinical School of Nanjing, Second Military Medical University, Nanjing, Jiangsu, China; 7 Department of Experimental Pathology, Beijing Institute of Radiation Medicine, Beijing, China; 8 The First Affiliated Hospital of Guangxi Medical University, Nanning, Guangxi, China; 9 Liver Cancer Institute at Fusui County, Guangxi, China; 10 Disease Prevention and Control Center at Haimen County, Jiangsu, China; Nanjing Medical University, CHINA

## Abstract

**Objective:**

*HJURP* (Holliday Junction-Recognizing Protein) plays dual roles in DNA repair and in accurate chromosome segregation during mitosis. We examined whether the single nucleotide polymorphisms (SNPs) of *HJURP* were associated with the risk of occurrence of hepatocellular carcinoma (HCC) among chronic hepatitis B virus (HBV) carriers from well-known high-risk regions for HCC in China.

**Methods:**

Twenty-four haplotype-tagging SNPs across *HJURP* were selected from HapMap data using the Haploview software. We genotyped these 24 SNPs using the using Sequenom's iPLEX assay in the Fusui population, consisting of 348 patients with HCC and 359 cancer-free controls, and further investigated the significantly associated SNP using the TaqMan assay in the Haimen population, consisting of 100 cases and 103 controls. The genetic associations with the risk of HCC were analyzed by logistic regression.

**Results:**

We observed an increased occurrence of HCC consistently associated with A/C or C/C genotypes of the non-synonymous SNP rs3771333 compared with the A/A genotype in both the Fusui and Haimen populations, with a pooled odds ratio 1.82 (95% confidence interval, 1.33–2.49; *P* = 1.9 × 10^−4^). Case-only analysis further indicated that carriers of the at-risk C allele were younger than those carrying the A/A genotype (*P* = 0.0016). In addition, the expression levels of HJURP in C allele carriers were lower than that in A/A genotype carriers (*P* = 0.0078 and 0.0010, for mRNA and protein levels, respectively).

**Conclusion:**

Our findings suggest that rs3771333 in *HJURP* may play a role in mediating the susceptibility to HCC among Chinese.

## Introduction

There is a high incidence of hepatocellular carcinoma (HCC) in China and other Asia-Pacific region but a low incidence in the United States and Europe. The development of HCC is a multifactorial and polygenic event with both environmental and genetic components. The environmental factors include chronic infection with hepatitis B (HBV) or C virus (HCV), aflatoxin exposure, cigarette smoking, alcohol consumption, cirrhosis, male gender and family history of HCC [1,2]. Chronic HBV infection contributes to most of HCC cases throughout the world [2], but only about one fifth of chronic HBV carriers suffer from HCC in their lifetime [1], suggesting that other risk factors may contribute to the outcome of chronic HBV infection.

Segregation analysis of familial HCC suggests interaction between HBV infection and genetic factors [3,4]. Recently, genome-wide association studies (GWAS) among Chinese determined that single-nucleotide polymorphisms (SNPs) at 1p36.22, 2q32.2–32.3, 6p21.3 and 21q21.3 are associated with HBV related HCC [5–7]. The identification of susceptibility genes contributing to HCC would help to predict individual and population risks of HCC and clarify pathophysiologic mechanisms relevant to this malignancy.

In humans, chronic HBV infection causes an ongoing inflammatory response that results in oxidative damage to the DNA of liver cells, and such damage can be converted to double-strand breaks (DSBs) during hepatocyte regeneration in response to cell death of liver tissue [8,9]. As one of the most serious forms of DNA damage that can occur in the genome, DSBs accumulation results in genomic instability and HBV integration and contributes to the progress of HCC among other types of cancer [10]. DSBs can be repaired without error by the homologous recombination (HR), commonly involving strand invasion of a sister chromatid, or by error-prone mechanisms such as nonhomologous end joining (NHEJ) or single-strand annealing [11]. The HR pathway in DSBs repair provides an efficient and faithful pathway of repair, especially in replicating cells, in which it plays a major role in maintenance of genome stability and tumor suppression [12]. HR defects can promote genomic instability and contribute to carcinogenesis [13].

Holliday junction-recognizing protein (*HJURP*), first annotated as human fetal liver expressing gene 1 (*hFLEG1*, GenBank accession number AB101211) is involved in the HR repair of DSBs through involved in ATM signaling, and through interaction with human MSH5 and NBS1, which is a part of the MRE11-RAD50-NBS1 (MRN) DNA repair complex [14]. MRN possesses critical end-bridging and endonucleolytic activities for the HR repair of DSBs [15]. The ATM protein kinase is activated by DSBs through the MRN DNA repair complex and orchestrates signaling cascades that initiate the DNA damage response [16]. Treatment of cancer cells with small interfering RNA (siRNA) against *HJURP* has caused abnormal chromosomal fusions and led to genomic instability [14], a common feature of human HCC [17].

In addition to the role in the HR repair of DNA DSBs, HJURP also participates in the accurate chromosome segregation during mitosis. HJURP has recently been reported to interact with CENP-A for the purpose of localizing CENP-A and loading new CENP-A nucleosomes on the centromere [18–22]. CENP-A is the key determinant of centromere formation and kinetochore assembly, which regulate the complex job of attaching chromosomes to the mitotic spindle, ensuring that those attachments are correct, signaling to delay mitotic progression if they are not, and regulating the movements of the chromosomes towards the spindle poles in anaphase [23]. *HJURP* down-regulation results in a dramatic loss of CENP-A from centromeres, likely impacting on kinetochore assembly and microtubule attachment, which can explain defects in chromosome segregation that ensue during mitosis [18–20]. Defects in chromosome segregation during mitosis result in aneuploidy, promote tumorigenesis [24] and are a common cytogenetic feature of cancer cells including HCC [25,26]. Given the above-mentioned important roles of *HJURP* in carcinogenesis, it is not surprising that *HJURP* is dysregulated in tissues of several types of cancer [14] including HCC (our own unpublished data of mRNA expression profile).

On the basis that *HJURP* is pivot in the HR repair of DSBs and the accurate chromosome segregation during mitosis to suppress tumorigenesis, and that *HJURP* is dys-regulated in HCC, we hypothesize that the *HJURP* gene may be a biological candidate susceptibility gene for HCC. It is expected that functional SNPs within *HJURP* could result in genotype-dependent difference in susceptibility to HCC. To cover the hidden causative SNPs in the present study, we genotyped 24 haplotype-tagging SNPs (htSNPs) across a 23-kilobase (kb) region spanning the *HJURP* gene, and performed association analyses in a Chinese HCC case-control population. We then attempted to replicate our association in an additional Chinese case-control population. At last, we investigated the relationship between the at-risk SNP (rs3771333) and the mRNA and protein expression of *HJURP* in tissues or cell lines.

## Materials and Methods

### Ethics statement

This study was performed with the approval of the Ethical Committee of Beijing Institute of Radiation Medicine (Beijing, China). At recruitment, written informed consent was obtained from all participants involved in this study.

### Study participants

This study included two independent Chinese case-control populations (Fusui population and Haimen population), totally consisting of 448 chronic HBV carriers with HCC (cases) and 462 chronic HBV carriers without HCC (controls; S1 Table). All the participants were unrelated adult Han Chinese.

#### Fusui population

This population consists of 348 cases who were recruited between July 2002 and January 2008 at the Guangxi Cancer Hospital (Nanning, China), and 359 control subjects who were randomly selected from a community cancer screening program for early detection of cancer conducted in the same regions during the same time period as the HCC cases were enrolled. All the subjects were residents in Fusui county and the surrounding regions at Guangxi province, a well-known high-risk region for HCC located in southern China. The enrollment criterion has been described in detail previously [5]. Briefly, the cases were chronic HBV carriers, newly diagnosed, previously untreated (chemotherapy or radiotherapy), pathologically confirmed, and proved not to have other cancers. The diagnosis of HCC was made by either positive histologic findings or an elevated serum α-fetoprotein level (≥ 400 ng/mL) combined with at least one positive image on angiography, sonography, and/or high-resolution contrast computed tomography. The controls were also chronic HBV carriers, and the selection criteria for them included no individual history of cancer and frequency matching to the cases on sex and age (± 5 years). The HBV carriers are subjects positive for both hepatitis B surface antigen (HBsAg) and antibody immunoglobulin G to hepatitis B core antigen (anti-HBcAg) for at least 6 months. Subjects were considered smokers if they smoked up to 1 year before the date of cancer diagnosis for cases or up to the date of interview for controls. Information was collected on the number of cigarettes smoked per day, the age at which the subjects started smoking, and the age at which ex-smokers stopped smoking. An alcohol drinker was defined as someone who consumed alcoholic beverages at least once per week for ≥ 6 months. All subjects included in this study were negative for antibodies to HCV, hepatitis D virus, or human immunodeficiency virus; and had no other types of liver disease, such as autoimmune hepatitis, toxic hepatitis, and primary biliary cirrhosis or Budd—Chiari syndrome.

#### Haimen population

This population consisting of 100 cases and 103 controls, was recruited from Haimen county at Jiangsu province, another well-known high-risk region for HCC located in eastern China. The enrollment criteria were identical to that in the Fusui population. The cases were consecutively recruited at the Center of Disease Control in Haimen County (Haimen, China) between August 2005 and July 2007. The response rate for case patients was 90%. A total of 103 cancer-free subjects were recruited as controls in the same regions during the same time period as the cases were collected. The response rate for control subjects was 92%.

**Healthy controls (non-HBV carriers)**: This control population totally consists of 280 non-HBV carriers as previously described [5]. Briefly, all the subjects were unrelated ethnic adult Han Chinese and randomly selected by community-based screening for early detection of cancer or non-infectious diseases conducted in Guangxi or Jiangsu province during between August 2003 and May 2009. The inclusion criteria for them included no individual history of cancer and negative for both HBsAg and anti-HBcAg. The male/female ratio and the mean age of these healthy controls is 2.3 (201/82) and 59.3 years (SD, 10.9), respectively.

### htSNP selection

Using HapMap Public Release #27 data (merged phases II+III) for the Han Chinese in Beijing, China (CHB), we constructed haplotype blocks across the *HJURP* gene plus ~3-kb flanking regions using the default ‘confidence interval’ option implemented in the Haploview software (version 4.2; http://www.broad.mit.edu/mpg/haploview/). An *r*^*2*^ threshold of 0.8 was set, and SNPs with minor allele frequency (MAF) < 0.05, call rate < 75% and Hardy-Weinberg equilibrium (HWe) *P* value < 0.01 were excluded. Nine SNPs in coding-regions SNPs were included as htSNPs using the forced inclusion option. Totally, 24 htSNPs were selected using Haploview (S1 Fig and S2 Table).

### Polymorphism genotyping

Genomic DNA was extracted from peripheral blood leukocytes of 5 mL whole blood of all participants by standard procedures. The 24 htSNPs in the Fusui population were genotyped using the Sequenom MassARRAY system according to the manufacturer’s instructions [27]. The MassARRAY system is based on multiplexed polymerase chain reaction (PCR) and single-base primer extension technology, and the mass of the extension products is measured with a matrix-assisted laser desorption ionization time-of-flight (MALDI-TOF) mass spectrometry to be correlated with a specific genotype. To ensure genotyping quality, we genotyped 5% randomly selected duplicate samples and 4 blanks in each 384-well plate and the concordance rate was greater than 99%. For primers and probes used for these SNPs, see S3 Table.

The significantly associated SNP (rs3771333) went forward to be genotyped in the Haimen population and cell lines using TaqMan MGB technology (Assay ID: C_25474467_10; Applied Biosystems, Foster City, CA, USA) according to the manufacturer’s protocol. For quality control, we genotyped by direct DNA sequencing a 15% masked, random sample set of the Haimen population and all results were 100% concordance. The experimenters conducted the genotyping in a blind manner and did not know the case-control status.

### Semiquantitative PCR

RNA templates were extracted from Epstein-Barr virus (EBV)-transformed blood lymphocyte cell lines derived from 53 unrelated healthy Chinese individuals using the RNeasy miniprep kit (Qiagen)[5]. Two micrograms of total RNA was reverse transcribed in a 20 μL reaction volume using the reverse transcriptase protocol (iScript, BioRad). The resulting cDNA was diluted 20 times in sterilized ultrapure water. Semiquantitative PCR experiments were carried out with the following synthesized *HJURP*-specific primers and with *GAPDH*-specific primers as an internal normalization control: *HJURP*, 5’-CAGAAGAGAGAGGAGAGAACACG-3’ and 5’-GAGGTTATCTCTGATGGAACCAA-3’; GAPDH, 5’-CCAGAACATCATCCCTGC-3’ and 5’-GGAAGGCCATGCCAGTGAGC-3’. PCR reactions were optimized for the number of cycles to ensure product intensity within the logarithmic phase of amplification.

### Immunohistochemistry (IHC)

Sixty HBV-related HCC patients were selected randomly from the 348 cases from ‘Fusui population’, of whom the paraformaldehyde-fixed paraffin-embedded tumor tissues and paired adjacent non-tumor liver tissues (n = 60) were analyzed for protein expression of HJURP with rabbit polyclonal Anti-HJURP (HPA008436, Sigma Aldrich). The IHC was performed and the signals were scored as previously described [5].

### Statistical Analysis

The *χ*^*2*^ test was performed to compare sex, age, smoking and drinking status between cases and controls. The unpaired *t* test was used to analyze the differences of mean age and smoking level between cases and controls. Frequencies of genotype and allele for the SNPs were determined by gene counting. The significance of deviations from HWe was tested using the random-permutation procedure implemented in the Arlequin software (http://lgb.unige.ch/arlequin/). To avoid spurious associations due to a small number of homozygous individuals, we used the dominant model to evaluate associations between the genotypes and HCC risk. The odds ratios (OR) and 95% confidence intervals (CI) were calculated by multivariate logistic regression analyses, adjusted for age, sex, smoking and drinking status, smoking level and family history, where it was appropriate.

The comparison between the rs3771333 C allele carriers and the A/A carriers were conducted by a *t* test and Mann-Whitney *U* test, for *HJURP* mRNA and protein levels, respectively. The differences of the protein levels between the tumors and paired adjacent non-tumors were assessed by a Wilcox test.

A *P* value of < 0.05 after Bonferroni correction was used as the criterion of statistical significance for SNP association, and all statistical tests were two sided. These analyses were performed using the SPSS software (version 13.0; SPSS Inc, Chicago, IL).

## Results

### Demographic and clinical characteristics

The selected characteristics of patients with HCC and control subjects in the Fusui and Haimen populations are shown in S1 Table. Over all, there were no significant differences between patients with HCC and control subjects in terms of sex, smoking and drinking status, and smoking level. However, in the Fusui population, the mean age of cases was higher than that of controls (*P* = 1.2 × 10^−6^), and more cases showed higher age (*P* = 3.0 × 10^−5^) and positive history of HCC in their first-degree biological relatives (*P* = 1.2 × 10^−5^)[5]. In the Haimen population, the mean age of cases was higher than that of controls (*P* = 8.3 × 10^−4^). In the pooled population with data derived from the two case-control sets (consisting of a total of 448 cases and 462 controls), the mean age of cases remained higher than that of controls (*P* = 2.4 × 10^−5^), and more cases were older (*P* = 1.2 × 10^−4^) and had family history of HCC (*P* = 4.8 × 10^−6^).

### rs3771333 associated with the risk of HCC in both the Fusui and Haimen populations

The ~23-kb genome region investigated in the present study (chr2:234407760- chr2:234430852), which contains the *HJURP* gene plus ~3-kb flanking regions, is large enough to cover all the four haplotype blocks across the *HJURP* gene (S1 Fig). The selected htSNPs and SNPs captured by them are given in S2 and S3 Tables.

The genotyping results of the 24 htSNPs in the Fusui population are summarized in Table 1. *P* value, OR and 95% CI were not available for rs2302154 due to monomorphism. The observed genotype frequencies of the remaining 23 polymorphisms conformed to the HWe in both cases and controls (all unadjusted *P* values > 0.01). An increased risk of HCC was found to be associated with the rs3771333 A/C or C/C genotypes, with the OR being 1.74 (95% CI, 1.23–2.46; *P* = 0.0017), compared with the A/A genotype (Table 1). The association withstood Bonferroni correction for multiple comparisons. rs3771333 (Glu568Asp, based on the sequence of NP_060880.3) is a non-synonymous SNP in exon 8 of the *HJURP* gene. For the remaining 22 SNPs, we found no significant association with the risk of HCC (all adjusted *P* values > 0.05), but we noticed that rs529963 in *HJURP* showed marginal association (uncorrected *P* value = 0.0037) with HCC risk (Table 1), and was in strong linkage disequilibrium (LD) with rs3771333 (*r*^*2*^ = 0.79).

**Table 1:** Summary of the association results of the 24 polymorphisms in the Fusui population.

					Fusui population			
SNP	Chr	Position^a^	Type	Alleles^b^	348 cases^c^	359 controls^c^	OR (95% CI)	*P* value
rs11563233	2	234407760	Intronic	T/A	288/58/2	291/66/2	1.17 (0.78–1.74)	0.45
rs213553	2	234410714	3’ UTR	G/T	112/169/67	108/181/70	1.18 (0.85–1.64)	0.33
rs213554	2	234412006	Intronic	T/C	88/187/73	79/188/92	1.27 (0.88–1.82)	0.2
rs3755317	2	234412104	Intronic	T/C	201/133/14	204/132/23	1.01 (0.74–1.38)	0.95
rs3771340	2	234413440	Intronic	G/A	163/149/36	159/149/51	1.18 (0.86–1.60)	0.3
rs965835	2	234413553	Intronic	A/G	95/171/82	102/177/80	1.00 (0.71–1.41)	1
rs213555	2	234413890	Intronic	A/G	213/119/16	216/115/28	1.15 (0.84–1.58)	0.38
rs3178178	2	234413997	Non_synonymous_coding	A/G	179/145/24	187/146/26	0.96 (0.71–1.30)	0.79
rs12582	2	234414093	Non_synonymous_coding	G/A	190/139/19	191/145/23	0.92 (0.68–1.25)	0.61
rs3771333	2	234414461	Non_synonymous_coding	A/C	238/103/7	278/74/7	1.74 (1.23–2.46)	0.0017
rs3821238	2	234414519	Non_synonymous_coding	C/G	178/146/24	187/146/26	0.95 (0.70–1.29)	0.74
rs3732215	2	234415281	Non_synonymous_coding	C/G	170/149/29	177/138/44	1.07 (0.78–1.45)	0.68
rs3806589	2	234415570	Non_synonymous_coding	G/A	192/131/25	210/132/17	0.82 (0.60–1.12)	0.22
rs6431641	2	234416320	Intronic	G/T	287/56/5	286/67/6	1.15 (0.78–1.70)	0.49
rs213556	2	234418146	Intronic	A/G	332/16/0	347/12/0	0.79 (0.36–1.74)	0.55
rs28900712	2	234421363	Intronic	G/A	326/22/0	347/12/0	0.46 (0.22–0.96)	0.035
rs529963	2	234423239	Synonymous_coding	C/T	229/111/8	269/81/9	1.71 (1.23–2.38)	0.0037
rs2286430	2	234425964	Non_synonymous_coding	G/A	167/149/32	171/140/48	1.09 (0.80–1.48)	0.59
rs626110	2	234427219	Intronic	A/T	83/181/84	110/158/91	0.74 (0.52–1.04)	0.082
rs2302154	2	234427876	Non_synonymous_coding	A/G	348/0/0	359/0/0	-	-
rs13406453	2	234430711	Upstream	T/C	310/38/0	330/27/2	0.76 (0.45–1.29)	0.3
rs6754410	2	234430761	Upstream	G/T	231/103/14	235/115/9	1.01 (0.73–1.40)	0.93
rs528971	2	234430820	Upstream	A/G	161/159/28	166/143/50	0.93 (0.68–1.26)	0.62
rs686802	2	234430852	Upstream	T/A	307/39/2	322/35/2	0.90 (0.55–1.48)	0.68

Chr, chromosome; OR, odds ratio; CI, confidence interval; UTR, untranslated region.

^a^Genomic position (NCBI Build 36).

^b^Major allele / minor allele.

^c^Number of major homozygotes / number of heterozygotes / number of minor homozygotes.

*P* values, ORs and 95% CIs were calculated under dominant model by logistic regression while adjusting for age, sex, status of smoking and drinking, smoking level and family history of hepatocellular carcinoma in the Fusui population. *P* value, OR and 95% CI were not available for rs2302154 in the Fusui population due to monomorphism. rs3771333 showed significant association in the Fusui population, therefore was further genotyped in the Haimen population.

However, the multiple logistic regression analysis showed that the association of rs529963 was not independent of the most significantly associated SNP rs3771333 (residual *P* = 0.47; S4 Table). Furthermore, haplotype analysis of rs3771333 and rs529963 showed that the haplotype C-T was significantly associated with the risk of occurrence of HCC (*P* = 0.0034; S5 Table), but inferior to rs3771333 in terms of the significance of association. We also searched for the potential associations between the haplotypes and the risk of HCC in each of the four haplotype blocks. However, in none of the four blocks did the haplotype analysis show any significant associations (*P* > 0.01; S6 Table). We went forward to genotype rs3771333 in the Haimen population. Consistently, rs3771333 showed significant association in this independent case-control set, with the OR being 2.39 (95% CI, 1.05–5.44; *P* = 0.036). In the pooled population, this association yielded a *P* value of 1.9 × 10^−4^, with the OR being 1.82 (95% CI, 1.335–2.49; Table 2).

**Table 2:** Association results of rs3771333.

Population	Genotypes	Cases, N (%)	Controls, N (%)	OR (95% CI)	*P* value
Fusui					
	A/A	238 (68.4)	278 (77.4)	1	
	A/C	103 (29.6)	74 (20.6)	1.77 (1.24–2.53)	0.0017
	C/C	7 (2.0)	7 (2.0)	1.45 (0.49–4.25)	0.49
	A/C + C/C	110 (31.6)	81 (22.6)	1.74 (1.23–2.46)	0.0016
Haimen					
	A/A	79 (79.0)	92 (89.3)	1	
	A/C	21 (21.0)	10 (9.7)	3.00 (1.25–7.23)	0.013
	C/C	0 (0.0)	1 (1.0)	-	-
	A/C + C/C	21 (21.0)	11 (10.7)	2.39 (1.05–5.44)	0.036
Pooled					
	A/A	317 (70.8)	366 (80.1)	1	
	A/C	124 (27.7)	82 (17.9)	1.90 (1.37–2.63)	1.0 × 10^−4^
	C/C	7 (1.5)	9 (2.0)	1.05 (0.38–2.88)	0.92
	A/C + C/C	131 (29.2)	91 (19.9)	1.82 (1.33–2.49)	1.9 × 10^−4^

OR, odds ratio; CI, confidence interval. *P* values, ORs and 95% CIs were calculated under dominant model by logistic regression while adjusting for age, sex, status of smoking and drinking, smoking level, family history of hepatocellular carcinoma and population, where it was appropriate.

### Stratification analysis

The association between rs3771333 and the risk of HCC was further examined with stratification by age, sex, status of smoking and drinking, pack-years of smoking, and family history. In the Fusui population, the susceptibility to HCC associated with the rs3771333 A/C or C/C genotypes appeared to be more pronounced in chronic HBV carriers who were males, younger, nonsmokers, with lower level of smoking, nondrinkers, or with negative family history (Table 3). However, these differences could be attributed to chance (all *P* values > 0.05, test for homogeneity; Table 3), indicating that these confounding factors had no modification effect on the risk of HCC related to rs3771333 in this population. The results were similar in the Haimen population and the pooled population. We found no appreciable variation of the effect across the subgroups stratified by age (heterogeneity test *P* = 0.13) in the pooled population; however, a case-only analysis resulted in an estimate that the mean ages at diagnosis (± SD, years) were 44.7 (± 10.6) years for carriers of the at-risk C allele, 3.5 years younger than carriers of the A/A genotype with mean ages at diagnosis 48.2 (± 10.8) years (*P* = 0.0016).

**Table 3:** Risk of hepatocellular carcinoma associated with rs3771333 by potential risk factors in the Fusui, the Haimen and the pooled Populations.

	Fusui population					Haimen population					Pooled population				
Variables	A/A ^a^	A/C + C/C ^a^	OR (95% CI) ^b^	*P* ^b^	*P* ^c^	A/A ^a^	A/C + C/C ^a^	OR (95% CI) ^b^	*P* ^b^	*P* ^c^	A/A ^a^	A/C + C/C ^a^	OR (95% CI) ^b^	*P* ^b^	*P* ^c^
Sex					0.53					0.48					0.33
Male	206/240	98/70	1.82 (1.24–2.65)	0.0018		64/69	16/6	2.93 (1.07–8.02)	0.036		270/309	114/76	0.51 (0.36–0.77)	1.6× 10^−4^	
Female	32/38	13/11	1.32 (0.51–3.34)	0.56		16/19	4/5	1.61 (0.36–7.10)	0.53		48/57	17/16	1.42 (0.64–3.15)	0.38	
Age					0.029					0.051					0.56
≤ 44	105/171	62/58	1.89 (1.20–2.96)	0.0059		15/25	9/1	20.4 (1.91–217.88)	0.012		120/196	71/59	2.11 (1.37–3.25)	6.6× 10^−4^	
> 44	133/107	48/23	1.72 (0.96–3.07)	0.067		64/63	12/9	1.37 (0.53–3.53)	0.52		197/170	60/32	1.68 (1.03–2.73)	0.037	
Smoking status					0.53					0.38					0.44
Nonsmoker	156/163	68/54	1.83 (1.16–2.90)	0.0094		45/54	13/6	2.91 (0.99–8.52)	0.051		201/217	81/60	2.00 (1.32–3.05)	0.0010	
Smoker	82/115	42/36	1.71 (0.99–2.93)	0.051		34/34	8/4	2.17 (0.54–8.63)	0.27		116/149	50/40	1.65 (1.01–2.69)	0.43	
Smoking level (pack-year)					0.48					0.086					0.42
≤ 19	39/62	22/18	2.48 (1.12–5.52)	0.025		8/14	1/6	11.4 (0.82–160.66)	0.07		47/76	23/24	1.93 (0.92–4.07)	0.084	
> 19	43/53	20/18	1.39 (0.65–2.97)	0.39		16/20	3/2	0.22 (0.02–2.33)	0.21		59/73	23/20	1.07 (0.51–2.24)	0.85	
Drinking status					0.49					0.70					0.28
Nondrinker	178/202	78/60	1.65 (1.09–2.48)	0.017		54/62	13/8	1.80 (0.67–4.82)	0.24		232/264	91/68	1.71 (1.18–2.48)	0.0049	
Drinker	60/76	32/21	2.04 (1.05–3.93)	0.035		25/26	8/2	3.59 (0.55–23.34)	0.18		85/102	40/23	2.11 (1.16–3.83)	0.014	
First-family history					0.27				0.33						0.10
Negative	194/260	89/77	1.68 (1.16–2.43)	0.0060		79/67	10/17	2.07 (0.88–4.88)	0.096		273/327	99/94	1.72 (1.23–2.40)	0.0016	
Positive	34/17	19/3	3.85 (0.87–17.0)	0.075		9/12	0/4	-	1		43/29	19/7	4.96 (1.27–19.3)	0.021	

CI, confidence interval; OR, odds ratio. The number of genotyped samples varies because of genotyping failure for some individuals.

^a^ Number of cases / number of controls.

^b^ ORs (95% CIs) and *P* were calculated by logistic regression with the A/A genotype as the reference group, and were adjusted for age, sex, status of smoking and drinking, smoking level, family history of hepatocellular carcinoma and population, where appropriate within the strata.

^c^ For difference in ORs within each stratum (*i*.*e*. *P* value for homogeneity).

### Association between rs3771333 genotypes and expression of HJURP mRNA or protein

**Fig 1:**
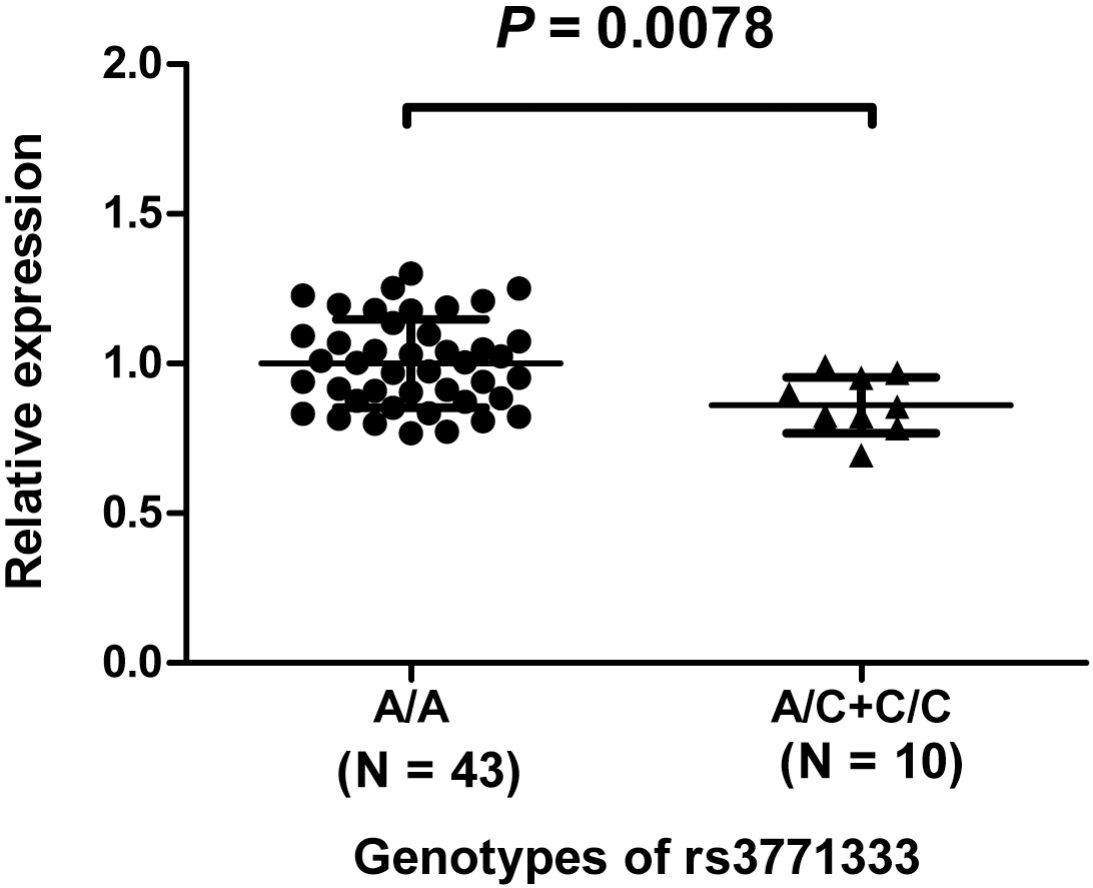
Semiquantitative PCR for *HJURP*. Expression of *HJURP* for the three different genotypes of rs3771333 was measured in RNA from Epstein-Barr virus (EBV)-transformed blood lymphocyte cell lines derived from 53 unrelated Chinese individuals. Forty-three individuals carry A/A, 9 carry A/C and 1 carrys C/C genotype. Normalization for mRNA quantity was performed with human *GAPDH* control primers for each sample. The horizontal bars indicate the mean values for each genotype, with the mean value of A/A carriers designated as 1. We found that the expression of *HJURP* mRNA in C allele carriers had a lower level compared with the A/A carriers (*P* = 0.0078; *t* test).

We further assessed the abundance of *HJURP* mRNA by semiquantitative PCR in EBV-transformed blood lymphocytes from 53 healthy individuals [5]. Expression of *HJURP* mRNA was markedly lower in the at-risk C allele carriers (*P* = 0.0078; Fig 1). By IHC, we detected greater expression of HJURP protein in HCC tissues than in paired adjacent non-tumor liver tissues (N = 60, *P* = 0.0010, Table 4). Furthermore, there was significant association between rs3771333 genotypes and expression of HJURP protein in the adjacent tissues, with the at-risk C allele carriers having lower HJURP than the A/A carriers (*P* = 0.024), but not in the HCC tissues (*P* = 0.64, Table 4).

**Table 4:** Protein expression of HJURP by immunohistochemical staining in 20 HCC tissues and paired adjacent non-tumor tissues.

	rs3771333	HJURP expression levels, N			*P* value	
		Negative	Low	High	A/C + C/C *vs*. A/A	Tumor *vs*. Adjacent
Tumor					0.64	0.0010
	A/A	0	15	30		
	A/C+C/C	0	6	9		
Adjacent					0.024	
	A/A	3	15	27		
	A/C+C/C	0	12	3		

Expression levels were classified into three groups (negative, low, and high expression) with scores of the immunohistochemistry signals. The differences of the protein levels between the tumors and paired adjacent non-tumors were assessed by a Wilcox test. The differences of the protein levels between the rs3771333 A/A genotype carriers and C allele carriers (A/C + C/C) were assessed by a Mann-Whitney *U* test.

## Discussion

In this study, we assessed the associations of htSNPs in the *HJURP* gene locus with the risk of occurrence of HBV-related HCC in two Chinese case-control populations. A non-synonymous SNP rs3771333 (Glu568Asp, based on the sequence of NP_060880.3) in exon 8 of the *HJURP* gene was significantly associated with the onset of HCC in the two case-control sets, separately and combined. Individuals carrying the rs3771333 C allele (A/C or C/C genotypes) have an increased risk of occurrence of HCC, compared to those with the A/A genotype. Subjects with the at-risk C allele tending to be younger than those with the A/A genotype by ages at diagnosis further supported a role of the *HJURP* polymorphism rs3771333 in the etiology of HCC. In addition, the mRNA and protein levels of HJURP in at-risk C allele carriers were lower than that in the A/A genotype carriers. To our knowledge, this is the first report of genetic association between the *HJURP* gene and risk of HCC (and any other cancer), confirming the initial hypothesis that *HJURP* may play a role in the pathogenesis of this malignancy.

In a separate study, we genotyped the rs3771333 in an additional healthy control population of Chinese origin, whom are unrelated non-HBV carriers (N = 280, S7 Table). The genotypes of this healthy control population were in HWe. The frequency of rs3771333 A/C or C/C genotype in these healthy controls was similar to that of chronic HBV carriers without HCC (i.e. controls; *P* = 0.79), but as expected significantly higher than that of chronic HBV carriers with HCC (i.e. cases; *P* = 0.010, S7 Table). These results suggest that rs3771333 represents a true SNP associated with HCC susceptibility, but not reflecting some other aspect of disease biology related to HCC risk, for example HBV infection.

In addition to rs3771333, another SNP rs529963 in *HJURP* showed evidence of association with HCC risk in the Fusui population, but the association of rs529963 did not significant when adjusted for the effect of rs3771333 (Table 1 and S4 Table). Haplotype analysis of these two SNPs (rs3771333 and rs529963) indicated that the haplotype C-T was significantly associated with HCC risk (S5 Table), but inferior to rs3771333 in terms of the significance of association. These results suggest that there might be a single susceptibility locus in *HJURP*, and the signal is only attributed to rs3771333.

We have used the software-package FASTSNP [28] to predict *in silico* functional consequences of the risk-associated htSNP (rs3771333) and the SNP captured by it (rs28900714). Although the non-synonymous SNP rs3771333 appears not to affect protein structure, this polymorphism is predicted to alter binding of an exonic splicing enhancer (ESE) SF2/ASF (ESEfinder program) or change the number of ESE binding site (RESCUE-ESE program). In contrast, rs28900714 is located in intron 4 of the *HJURP* gene and predicted to exert no known function.

To test the possibility that the difference in ESE binding of the two alleles of rs3771333 may be relevant for the expression of its host gene *HJURP*, we have conducted a preliminary analysis of EBV-transformed blood lymphocyte cell lines derived from unrelated Chinese individuals, and indeed, we found mRNA levels of *HJURP* were markedly lower in at-risk C allele carriers. Furthermore, there was significant association between rs3771333 genotypes and expression of HJURP protein in adjacent non-HCC tissues, with the at-risk C allele carriers having lower HJURP than the A/A carriers. These results are consistent with the idea that the *HJURP* gene can act as a tumor suppressor.

Although the exact mechanism by which *HJURP* polymorphism influences the susceptibility to HBV-related HCC requires further investigation, the genetic association between *HJURP* polymorphism and susceptibility to HCC is biologically plausible. Previous studies have provided compelling evidence that defective DSB repair accelerates HBV-related HCC and other types of liver cancer [10][29–31], and that genetic polymorphisms in DSB repair genes and other DNA repair genes may contribute to HCC susceptibility [32,33]. Acting as a critical factor in the DSB repair pathway, the *HJURP* gene has also been dys-regulated in HCC as shown in the present study and our unpublished expression profile data. Furthermore, *HJURP* down-regulation caused genomic instability and defects in chromosome segregation during mitosis [14,18,19,34], both of which being the common features of human HCC [17,35].

Given the role of *HJURP* in the development of HCC, together with the functional relevance of rs3771333 in modulation of HJURP expression, one might expect that individuals who carry the at-risk rs3771333 C allele, and thus have decreased HJURP mRNA and protein levels and subsequently lower capacity to maintain genomic stability and proper chromosome segregation during mitosis, may be at a higher susceptibility to developing HCC. Additional experiments will be required to further elucidate the functional role of *HJURP* polymorphisms in mediating the susceptibility to HBV-related HCC.

If rs3771333 is regarded as a risk factor for the development of HCC, then the population attributable fraction (PAF) can be calculated to indicate the degree of elevation in the risk of developing HCC which can be attributed to the susceptible effect of the rs3771333 A/C or C/C genotypes. PAF can be estimated with the formula of f(RR-1)/[1+f(RR-1)], where f is the population exposure rate, and RR is the relative risk (odds ratio)[36]. The PAF calculated by the RR (OR, 1.74 [95% CI, 1.23–2.46], 2.39 [95% CI, 1.05–5.44] and 1.82 [95% CI, 1.33–2.49]) combined with the frequency of the A/C or C/C genotypes (22.6%, 10.7% and 19.9%) indicates that 14.3% (95% CI, 4.9–24.8%), 12.9% (95% CI, 0.53–32.2%) and 14.0% (95% CI, 6.2–22.9%) of the elevation in the risk of developing HCC can be attributed to the susceptibility effect of the rs3771333 A/C or C/C genotypes in the Fusui population, the Haimen population and the pooled population, respectively. Based on the calculated PAF, the *HJURP* rs3771333 polymorphism is not suitable for risk prediction testing at present. Therefore, more susceptibility genetic loci and their interactions with other HCC risk factors are needed to be identified, and then the risk prediction of HCC occurrence may become more accurate and clinically usable.

In the present study, we observed significantly higher expression of HJURP protein in HCC tissues compared with paired adjacent non-tumor tissues. These results were not surprised because that increased HJURP may result from failed attempts to repair ongoing damage [37,38]. Alternatively, increased HJURP may indicate a block in chromosome segregation during mitosis, leading to hyper-activation of HJURP (and CENPA)[38,39]. In future studies, it is worthy to investigate whether or not the elevated expression of HJURP may serve as a useful molecular marker for predicting progression and prognosis and as a target for therapy in patients with HCC.

It has been reported that HJURP was markedly over-expressed in a broad range of human cancers. Therefore, whether or not the *HJURP* polymorphism rs3771333 also plays a similar role in different types of cancer warrants further investigation.

There are several potential limitations in the present study. First, the patients with HCC were recruited from hospitals, while the controls were selected from community residents. Therefore, the inherent selection bias cannot be completely ruled out. Second, although the highly significant association between *HJURP* and susceptibility to HCC is derived from a biologically based *a priori* hypothesis, our initial findings should be verified in other independent populations with large number of subjects. Without rigorous replication we cannot exclude the possibility that these findings are due only to chance. Third, although alcohol and tobacco abuse have been well-documented as risk factors of HCC development, there were no significant differences of these factors between cases and controls (S1 Table). The non-significant differences can probably be explained by the above mentioned two limitations, namely the possible selection bias and small sample size (which means decreased power). However, there may exist other explanations such that not every previously established causative factors show associations with HCC risk simultaneously in an individual population. For example, in a HBsAg-positive cohort with 3,931 participants from Taiwan, family history of HCC (but neither smoking habit nor alcohol consumption) showed a significant association with HCC risk with a multivariate adjusted hazard ratio 2.46 [40]. Similar phenomena were also observed in the present study and many other case-control studies [6,41,42], suggesting that in the present study of HBV carriers, family history of HCC probably is the major environmental risk factor for HCC, other than smoking habit or alcohol consumption.

## Conclusions

Our results show an association between *HJURP* polymorphism and risk of HBV-related HCC in two Chinese case-control populations, and provide support for the importance of *HJURP* in the pathogenesis of HBV-related HCC. If confirmed by other studies, knowledge of genetic factors contributing to the pathogenesis of the HCC as presented here may have implications for the cancer screening and treatment of this disorder in the future.

## Supporting Information

S1 Fig Linkage disequilibrium maps of SNPs in the *HJURP* gene locus. The data were derived from HapMap CHB population (Release #27; merged phases II+III). The value in each diamond is measured as *r*^*2*^ corresponding to the dark-to-white gradient. Dark diamonds without a number indicate that the value of *r*^*2*^ was 1. Twenty-four haplotype-tagging SNPs (htSNPs) selected using Haploview were outlined in red box. When selecting htSNPs, an *r*^*2*^ threshold of 0.8 was set, and SNPs with minor allele frequency < 0.05, call rate < 75% and Hardy-Weinberg equilibrium *P* value < 0.01 were excluded. Nine SNPs in coding-regions SNPs were included as htSNPs using the forced inclusion option.
(DOCX)

S1 Table Selected characteristics of patients with hepatocellular carcinoma and controls in the Fusui and Haimen populations. SD, standard deviation. *P* values are calculated by *t* test (2-sided) for means of age and smoking level, and *χ*^2^ test (2-sided) for other variables.
(DOCX)

S2 Table htSNPs in the genomic region covering *HJURP* and SNPs captured by htSNPs. SNP, single nucleotide polymorphism. htSNP, haplotype-tagging SNP.
(DOCX)

S3 Table Primers and probes used for genotyping assays. The 24 htSNPs were genotyped using the Sequenom MassARRAY system according to the manufacturer’s instructions. PCR conditions were as follows: 95°C for 15 min, followed by 45 cycles of 95°C for 30 s, 56°C for 1 min, then 72°C for 1.5 min, with a final hold of 72°C of 7 min. The extension were as follows: 94°C hold for 2 min, with 75 cycles of 94°C for 5 s, 52°C for 5 s and 72°C for 5 s.
(DOCX)

S4 Table Association results of rs529963 in the Fusui population. OR, odds ratio; CI, confidence interval. *P* values, ORs and 95% CIs were calculated under dominant model by logistic regression while adjusting for age, sex, status of smoking and drinking, smoking level and family history of hepatocellular carcinoma. ^a^ Values before adjustment for rs3771333. ^b^ Values after adjustment for rs3771333.
(DOCX)

S5 Table Association of estimated haplotypes for rs3771333 and rs529963 with HCC. The haplotype is in the order of rs3771333 and rs529963. ^a^ No correction was made for testing multiple alleles. ^b^ Rare haplotypes with less than 5% frequency were pooled.
(DOCX)

S6 Table Association of estimated haplotypes in the *HJURP* gene locus with hepatocellular carcinoma. The haplotype in block 1 is in the order of rs213554 and rs3755317. The haplotype in block 2 is in the order of rs965835, rs213555, rs3178178 and rs12582. The haplotype in block 4 is in the order of rs213556, rs28900712, rs529963, rs2286430, rs626110, rs2302154, rs13406453, rs6754410 and rs528971. ^a^No correction was made for testing multiple alleles. ^b^Threre is only one haplotype-tagging SNP (rs6431641) genotyped in block 3, therefore haplotype analysis did not performed in this block. ^c^ Rare haplotypes with less than 5% frequency were pooled.
(DOCX)

S7 Table The allele and genotype frequencies of rs3771333 in different populations. Healthy controls are non-HBV carriers negative for both hepatitis B surface antigen and antibody immunoglobulin G to hepatitis B core antigen.
(DOCX)
