# Supplementary figures and images for: A Non-Synonymous Single Nucleotide Polymorphism in the HJURP Gene Associated with Susceptibility to Hepatocellular Carcinoma among Chinese
Source: PLoS One. 2016 Feb 10;11(2):e0148618. doi: 10.1371/journal.pone.0148618 (PMC4749235; doi:10.1371/journal.pone.0148618)

**
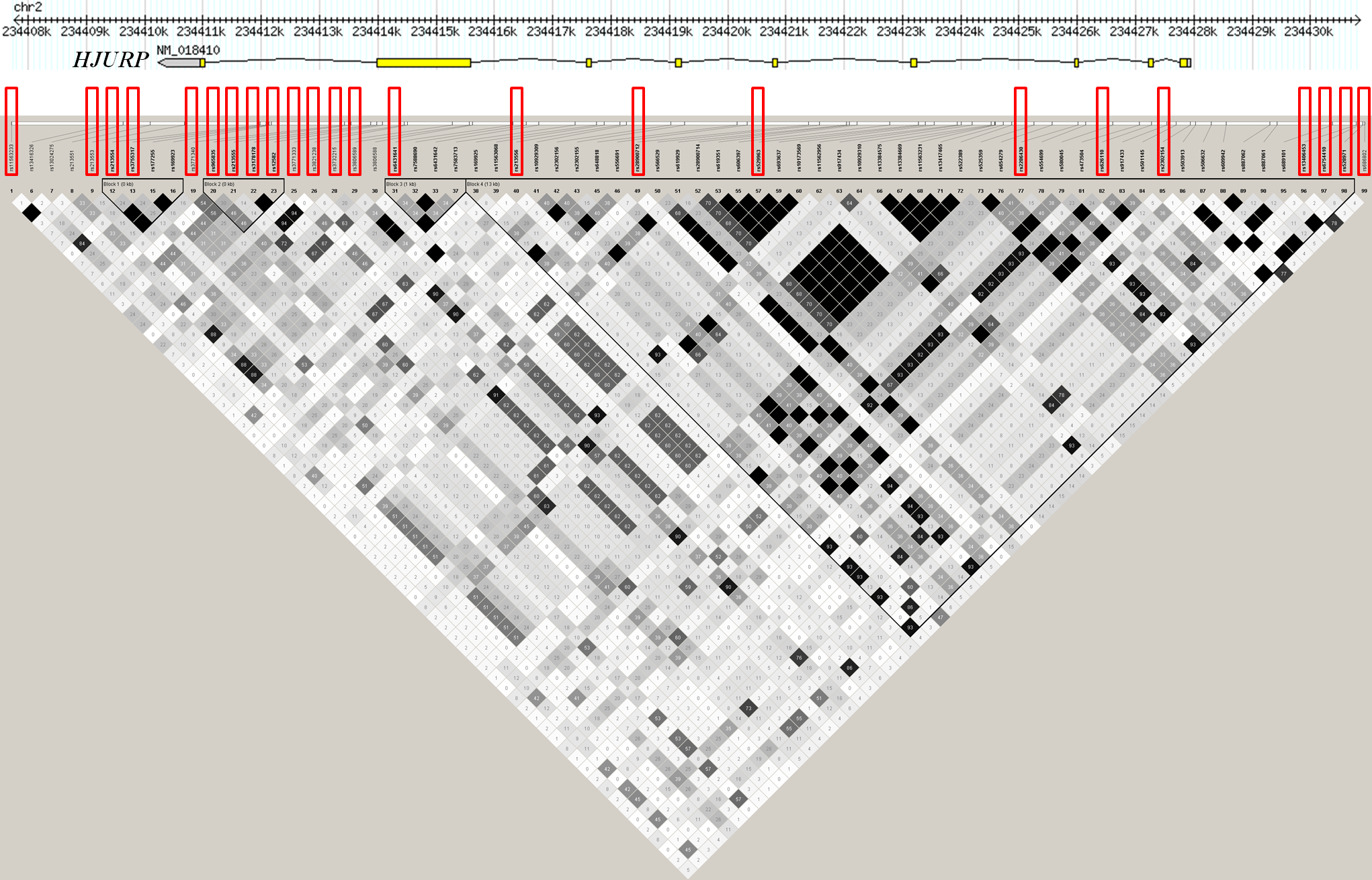
**

Supplement: S1 Fig — The data were derived from HapMap CHB population (Release #27; merged phases II+III). The value in each diamond is measured as r2 corresponding to the dark-to-white gradient. Dark diamonds without a number indicate that the value of r2 was 1. Twenty-four haplotype-tagging SNPs (htSNPs) selected using Haploview were outlined in red box. When selecting htSNPs, an r2 threshold of 0.8 was set, and SNPs with minor allele frequency < 0.05, call rate < 75% and Hardy-Weinberg equilibrium P value < 0.01 were excluded. Nine SNPs in coding-regions SNPs were included as htSNPs using the forced inclusion option. (DOCX) [file pone.0148618.s001.docx]
