# Supplementary material for: A Non-Synonymous Single Nucleotide Polymorphism in the HJURP Gene Associated with Susceptibility to Hepatocellular Carcinoma among Chinese
Source: PLoS One. 2016 Feb 10;11(2):e0148618. doi: 10.1371/journal.pone.0148618 (PMC4749235; doi:10.1371/journal.pone.0148618)
Supplement: S1 Table — SD, standard deviation. P values are calculated by t test (2-sided) for means of age and smoking level, and χ2 test (2-sided) for other variables. (DOCX) [file pone.0148618.s002.docx]

**S1 Table.** Selected characteristics of patients with hepatocellular carcinoma and controls in the Fusui and Haimen populations.

|  | Fusui population, N (%) | | |  | Haimen population, N (%) | | |  | Pooled population, N (%) | | |
| --- | --- | --- | --- | --- | --- | --- | --- | --- | --- | --- | --- |
| Variables | Cases | Controls |  |  | Cases | Controls |  |  | Cases | Controls |  |
|  | N = 348 | N = 359 | *P* value |  | N = 100 | N = 103 | *P* value |  | N = 448 | N = 462 | *P* value |
| Age, year |  |  |  |  |  |  |  |  |  |  |  |
| Mean (SD) | 45.8 (10.6) | 41.6 (12.1) | 1.2 × 10^-6^ |  | 51.8 (10.4) | 46.5 (11.9) | 8.3 × 10^-4^ |  | 47.2 (10.9) | 43.8 (12.7) | 2.4 × 10^-5^ |
| ≤ 44 | 167 (48.0) | 229 (63.8) | 3.0 × 10^-5^ |  | 24 (24.0) | 27 (26.2) | 0.75 |  | 191 (42.6) | 256 (55.4) | 1.2 × 10^-4^ |
| > 44 | 181 (52.0) | 130 (36.2) |  |  | 76 (76.0) | 76 (73.8) |  |  | 257 (57.4) | 206 (44.6) |  |
| Sex |  |  |  |  |  |  |  |  |  |  |  |
| Female | 45 (12.9) | 49 (13.6) | 0.83 |  | 20 (20.0) | 24 (23.3) | 0.61 |  | 65 (14.5) | 73 (15.8) | 0.64 |
| Male | 303 (87.1) | 310 (86.4) |  |  | 80 (80.0) | 79 (76.7) |  |  | 383 (85.5) | 389 (84.2) |  |
| Smoking status | |  |  |  |  |  |  |  |  |  |  |
| Nonsmoker | 224 (64.4) | 208 (57.9) | 0.090 |  | 58 (58.0) | 64 (62.1) | 0.57 |  | 282 (62.9) | 272 (58.9) | 0.22 |
| Smoker | 124 (35.6) | 151 (42.1) |  |  | 42 (42.0) | 39 (37.9) |  |  | 166 (37.1) | 190 (41.1) |  |
| Smoking level (pack-year) | |  |  |  |  |  |  |  |  |  |  |
| Mean (SD) | 20.5 (19.7) | 20.3 (19.0) | 0.93 |  | 15.3 (7.0) | 15.3 (10.2) | 1 |  | 19.2 (17.5) | 19.3 (17.7) | 0.96 |
| ≤ 19 | 61 (49.2) | 80 (53.0) | 0.55 |  | 20 (47.6) | 20 (51.3) | 0.83 |  | 81 (48.8) | 100 (52.6) | 0.52 |
| > 19 | 63 (50.8) | 71 (47.0) |  |  | 22 (52.3) | 19 (48.7) |  |  | 85 (51.2) | 90 (47.4) |  |
| Drinking status | |  |  |  |  |  |  |  |  |  |  |
| Nondrinker | 256 (73.6) | 262 (73.0) | 0.87 |  | 67 (67.0) | 73 (70.9) | 0.65 |  | 323 (72.1) | 335 (72.5) | 0.94 |
| Drinker | 92 (26.4) | 97 (27.0) |  |  | 33 (33.0) | 30 (29.1) |  |  | 125 (27.9) | 127 (27.5) |  |
| First-family history | |  |  |  |  |  |  |  |  |  |  |
| Negative | 292 (84.2) | 338 (94.4) | 1.2 × 10^-5^ |  | 84 (84.0) | 94 (91.3) | 0.14 |  | 376 (83.9) | 432 (93.5) | 4.8 × 10^-6^ |
| Positive | 56 (15.8) | 21 (5.6) |  |  | 16 (16.0) | 9 (8.7) |  |  | 72 (16.1) | 30 (6.5) |  |
