# Supplementary material for: A Non-Synonymous Single Nucleotide Polymorphism in the HJURP Gene Associated with Susceptibility to Hepatocellular Carcinoma among Chinese
Source: PLoS One. 2016 Feb 10;11(2):e0148618. doi: 10.1371/journal.pone.0148618 (PMC4749235; doi:10.1371/journal.pone.0148618)
Supplement: S2 Table — SNP, single nucleotide polymorphism. htSNP, haplotype-tagging SNP. (DOCX) [file pone.0148618.s003.docx]

**S2 Table.** htSNPs in the genomic region covering *HJURP* and SNPs captured by htSNPs.

| htSNPs | SNPs captured by htSNPs |
| --- | --- |
| rs11563233 | rs11563233, rs13024275 |
| rs213553 | rs213553 |
| rs213554 | rs213554, rs169923, rs177255 |
| rs3755317 | rs3755317, rs13416326 |
| rs3771340 | rs3771340 |
| rs965835 | rs965835 |
| rs213555 | rs213555, rs10929310 |
| rs3178178 | rs3178178 |
| rs12582 | rs12582 |
| rs3771333 | rs3771333, rs28900714 |
| rs3821238 | rs3821238 |
| rs3732215 | rs3732215 |
| rs3806589 | rs3806589, rs6431642, rs213551, rs7588690 |
| rs6431641 | rs6431641, rs7583713 |
| rs213556 | rs213556 |
| rs28900712 | rs28900712 |
| rs529963 | rs529963, rs606397, rs473504, rs13417465, rs522389, rs619351, rs566529,rs11563231, rs13384575, rs13384669, rs693637, rs10173569 |
| rs2286430 | rs2286430, rs887061, rs2302155, rs10929309, rs917433, rs917434 |
| rs626110 | rs626110, rs580045, rs619929, rs501145, rs556691, rs525359, rs654279, rs2302156 |
| rs2302154 | rs2302154 |
| rs13406453 | rs13406453, rs506632 |
| rs6754410 | rs6754410, rs11563068, rs3806588, rs11562956 |
| rs528971 | rs528971, rs169925, rs689101,rs609942, rs503913, rs887062, rs554699, rs648818 |
| rs686802 | rs686802 |
