# Supplementary material for: A Non-Synonymous Single Nucleotide Polymorphism in the HJURP Gene Associated with Susceptibility to Hepatocellular Carcinoma among Chinese
Source: PLoS One. 2016 Feb 10;11(2):e0148618. doi: 10.1371/journal.pone.0148618 (PMC4749235; doi:10.1371/journal.pone.0148618)
Supplement: S3 Table — The 24 htSNPs were genotyped using the Sequenom MassARRAY system according to the manufacturer’s instructions. PCR conditions were as follows: 95°C for 15 min, followed by 45 cycles of 95°C for 30 s, 56°C for 1 min, then 72°C for 1.5 min, with a final hold of 72°C of 7 min. The extension were as follows: 94°C hold for 2 min, with 75 cycles of 94°C for 5 s, 52°C for 5 s and 72°C for 5 s. (DOCX) [file pone.0148618.s004.docx]

**S3 Table. Primers and probes used for genotyping assays.**

| SNP | Primers | Sequences |
| --- | --- | --- |
| rs11563233 | Forward | 5'-ACGTTGGATGTTTGGACAGCATAGCAGCTC-3' |
|  | Reverse | 5'-ACGTTGGATGTTCCAAACTGTTCCCAGTCC-3' |
|  | Extension | 5'-AGGGACCCCGGATGGTGA-3' |
| rs213553 | Forward | 5'-ACGTTGGATGTGGGCGATAGAGAGAGACTG-3' |
|  | Reverse | 5'-ACGTTGGATGGGTTCTCTTTGATAGGCCTG-3' |
|  | Extension | 5'-ACAAATAACCCCCCCCC-3' |
| rs213554 | Forward | 5'-ACGTTGGATGATCTGAGGTTTCTCCTGGTC-3' |
|  | Reverse | 5'-ACGTTGGATGGGGCACTATCTGAGAAAAAG-3' |
|  | Extension | 5'-CCCCCTCAGAGCTCCTCTGCTCTGCT-3' |
| rs3755317 | Forward | 5'-ACGTTGGATGATGACCTCTCAAGAGTCTCC-3' |
|  | Reverse | 5'-ACGTTGGATGGGGCCGGACAGTTGTTTTTG-3' |
|  | Extension | 5'-GCAAGAGTCTCCTTCTCAG-3' |
| rs3771340 | Forward | 5'-ACGTTGGATGGCACACGCAGACTAAAACTC-3' |
|  | Reverse | 5'-ACGTTGGATGCTGCAGATATCTCAGTGCTC-3' |
|  | Extension | 5'-TCAGACTAAAACTCTAGCTGACA-3' |
| rs965835 | Forward | 5'-ACGTTGGATGCTGTGAGAATACTGTGCTCG-3' |
|  | Reverse | 5'-ACGTTGGATGTTTCTGGATTCCTGGTAGGG-3' |
|  | Extension | 5'-TATGTGCTCGTGGCTCTCCC-3' |
| rs213555 | Forward | 5'-ACGTTGGATGATGGTAGGAATCTTTGCGCC-3' |
|  | Reverse | 5'-ACGTTGGATGGCATTTGGGAGAAAGCTCAG-3' |
|  | Extension | 5'-TTTTCGCCACGTTTAGTGAATCTCA-3' |
| rs3178178 | Forward | 5'-ACGTTGGATGTCAACCAACAGAAACACACC-3' |
|  | Reverse | 5'-ACGTTGGATGTGGACAACACCGTCAGACC-3' |
|  | Extension | 5'-CAGAAACACACCTACCTC-3' |
| rs12582 | Forward | 5'-ACGTTGGATGTGTTGTCCACCCCATCTGAG-3' |
|  | Reverse | 5'-ACGTTGGATGGCAAAAAGACCCAGGCTATC-3' |
|  | Extension | 5'-GAATTGCCCTGGCGTCCG-3' |
| rs3771333 | Forward | 5'-ACGTTGGATGTCAGTGTCACCCAGCAAAAC-3' |
|  | Reverse | 5'-ACGTTGGATGTCGTAACGATTCCTTCCGTG-3' |
|  | Extension | 5'-GCTTTCAGTCCCAGATAAAGA-3' |
| rs3821238 | Forward | 5'-ACGTTGGATGTTTTGCTGGGTGACACTGAC-3' |
|  | Reverse | 5'-ACGTTGGATGAGCAGACATCTGACCTTCAC-3' |
|  | Extension | 5'-CACTGACTTTCTAAATATTCCA-3' |
| rs3732215 | Forward | 5'-ACGTTGGATGCCTGCTCTTATATCTGTGCC-3' |
|  | Reverse | 5'-ACGTTGGATGGCATCATCTCCACCAAAACG-3' |
|  | Extension | 5'-TCCATCTGTGCCTCCTCCTG-3' |
| rs3806589 | Forward | 5'-ACGTTGGATGCTTTTCTCCCAGGATACTGC-3' |
|  | Reverse | 5'-ACGTTGGATGAGATGAAGCTGGTTTCGCTG-3' |
|  | Extension | 5'-GGGTGATACTGCAGTCGTATCTCC-3' |
| rs6431641 | Forward | 5'-ACGTTGGATGAGAGGCAGATATGGCATCAG-3' |
|  | Reverse | 5'-ACGTTGGATGCTGGCTTTTGTGTCGTGTAG-3' |
|  | Extension | 5'-ATAAGTGCTCAAAAACGCATG-3' |
| rs213556 | Forward | 5'-ACGTTGGATGCAGGTACATCCACTAAGTCG-3' |
|  | Reverse | 5'-ACGTTGGATGTTCTTACTGTGCTGCTGACC-3' |
|  | Extension | 5'-AGTCGGTGGTCCACAAA-3' |
| rs28900712 | Forward | 5'-ACGTTGGATGTCCTTAGGTCCATGAATTTC-3' |
|  | Reverse | 5'-ACGTTGGATGTACTGGGCAGTACATCTATC-3' |
|  | Extension | 5'-GTCGGTCCATGAATTTCCCCAAC-3' |
| rs529963 | Forward | 5'-ACGTTGGATGTTGCACGGAGCCATCTGTC-3' |
|  | Reverse | 5'-ACGTTGGATGTTATTGGCTTCTGCACTTGG-3' |
|  | Extension | 5'-TGTCCGCGGGCTTCATGGA-3' |
| rs2286430 | Forward | 5'-ACGTTGGATGCCACAGCAAGGTTATTTGGC-3' |
|  | Reverse | 5'-ACGTTGGATGGGGTGGAAGACTAATAAAGG-3' |
|  | Extension | 5'-TACCTGGATCTCTCCTT-3' |
| rs626110 | Forward | 5'-ACGTTGGATGCACACTCTTCACCCGAATAC-3' |
|  | Reverse | 5'-ACGTTGGATGAAATGGCCACGCTGACCTAC-3' |
|  | Extension | 5'-TACTCAGAAGGTGGGGA-3' |
| rs2302154 | Forward | 5'-ACGTTGGATGTCCTATTTGAGTTTGTGGCG-3' |
|  | Reverse | 5'-ACGTTGGATGTGGTCGTCTTCCACGTCCTC-3' |
|  | Extension | 5'-GGGCTGGGTCCGATGCTGGGT-3' |
| rs13406453 | Forward | 5'-ACGTTGGATGACATAACCGAGTAGGTCAGG-3' |
|  | Reverse | 5'-ACGTTGGATGCCTCATATTGCCTTATGCTC-3' |
|  | Extension | 5'-GGCGCCAGGCTGTCTGC-3' |
| rs6754410 | Forward | 5'-ACGTTGGATGAGACCACTCCTCATATTGCC-3' |
|  | Reverse | 5'-ACGTTGGATGTCTTTAACTACCAGGCCCAG-3' |
|  | Extension | 5'-CCTAATATTGCCTTATGCTCAATTT-3' |
| rs528971 | Forward | 5'-ACGTTGGATGCTCCTCCCTTAATGCCATTC-3' |
|  | Reverse | 5'-ACGTTGGATGAGGAGTTTGCTTAGCAGGTC-3' |
|  | Extension | 5'-CTTAATGCCATTCTCTGTAA-3' |
| rs686802 | Forward | 5'-ACGTTGGATGCTCCTCCCTTAATGCCATTC-3' |
|  | Reverse | 5'-ACGTTGGATGAGGAGTTTGCTTAGCAGGTC-3' |
|  | Extension | 5'-CACCCTTGGCAGCCTAACC-3' |
