# Supplementary material for: A Non-Synonymous Single Nucleotide Polymorphism in the HJURP Gene Associated with Susceptibility to Hepatocellular Carcinoma among Chinese
Source: PLoS One. 2016 Feb 10;11(2):e0148618. doi: 10.1371/journal.pone.0148618 (PMC4749235; doi:10.1371/journal.pone.0148618)
Supplement: S4 Table — OR, odds ratio; CI, confidence interval. P values, ORs and 95% CIs were calculated under dominant model by logistic regression while adjusting for age, sex, status of smoking and drinking, smoking level and family history of hepatocellular carcinoma. a Values before adjustment for rs3771333. b Values after adjustment for rs3771333. (DOCX) [file pone.0148618.s005.docx]

**S4 Table.** Association results of rs529963 in the Fusui population.

| Polymorphisms | Cases, N (%)  (n = 348) | Controls, N (%)  (n = 359) | OR (95% CI) ^a^ | *P* value ^a^ | OR (95% CI) ^b^ | *P* value ^b^ |
| --- | --- | --- | --- | --- | --- | --- |
| rs529963 |  |  |  |  |  |  |
| C/C | 229 (65.8) | 269 (74.9) | 1.73 (1.20-2.51) | 0.0037 | 1.40 (0.55-3.57) | 0.47 |
| C/T | 111 (31.9) | 81 (22.6) |  |  |  |  |
| T/T | 8 (2.3) | 9 (2.5) |  |  |  |  |
| C/T + T/T | 119 (34.2) | 90 (25.1) |  |  |  |  |
