# Supplementary material for: A Non-Synonymous Single Nucleotide Polymorphism in the HJURP Gene Associated with Susceptibility to Hepatocellular Carcinoma among Chinese
Source: PLoS One. 2016 Feb 10;11(2):e0148618. doi: 10.1371/journal.pone.0148618 (PMC4749235; doi:10.1371/journal.pone.0148618)
Supplement: S5 Table — The haplotype is in the order of rs3771333 and rs529963. a No correction was made for testing multiple alleles. b Rare haplotypes with less than 5% frequency were pooled. (DOCX) [file pone.0148618.s006.docx]

**S5 Table.** Association of estimated haplotypes for rs3771333 and rs529963 with HCC.

| Haplotype | Cases, 2N (%)  (N = 348) | Controls, 2N (%)  (N = 359) | OR (95% CI) | *P* value ^a^ |
| --- | --- | --- | --- | --- |
| A-C | 563 (80.9) | 617 (85.9) | 1 |  |
| C-T | 111 (15.9) | 86 (12.0) | 1.72 (1.20 - 2.47) | 0.0034 |
| Rare | 22 (3.2) | 15 (2.1) | 1.37 (0.63 - 2.97) | 0.43 |
