# Supplementary material for: A Non-Synonymous Single Nucleotide Polymorphism in the HJURP Gene Associated with Susceptibility to Hepatocellular Carcinoma among Chinese
Source: PLoS One. 2016 Feb 10;11(2):e0148618. doi: 10.1371/journal.pone.0148618 (PMC4749235; doi:10.1371/journal.pone.0148618)
Supplement: S6 Table — The haplotype in block 1 is in the order of rs213554 and rs3755317. The haplotype in block 2 is in the order of rs965835, rs213555, rs3178178 and rs12582. The haplotype in block 4 is in the order of rs213556, rs28900712, rs529963, rs2286430, rs626110, rs2302154, rs13406453, rs6754410 and rs528971. aNo correction was made for testing multiple alleles. bThrere is only one haplotype-tagging SNP (rs6431641) genotyped in block 3, therefore haplotype analysis did not performed in this block. c Rare haplotypes with less than 5% frequency were pooled. (DOCX) [file pone.0148618.s007.docx]

**S6 Table.** Association of estimated haplotypes in the *HJURP* gene locus with hepatocellular carcinoma.

| Haplotype | Cases, 2N (%)  (N = 348) | Controls, 2N (%)  (N = 359) | OR (95% CI) | *P* value ^a^ |
| --- | --- | --- | --- | --- |
| Block 1 |  |  |  |  |
| T-T | 363 (52.2) | 346 (48.2) | 1 |  |
| C-T | 172 (24.7) | 194 (27.0) | 0.76 (0.57 - 1.02) | 0.068 |
| C-C | 161 (23.1) | 178 (24.8) | 0.88 (0.65 - 1.19) | 0.41 |
|  |  |  |  |  |
| Block 2 |  |  |  |  |
| G-A-G-A | 193 (27.7) | 197 (27.4) | 1 |  |
| G-A-A-G | 168 (24.1) | 185 (25.8) | 1.01 (0.72 - 1.41) | 0.97 |
| A-A-A-G | 184 (26.4) | 166 (23.1) | 1.28 (0.92 - 1.79) | 0.15 |
| A-G-A-G | 151 (21.7) | 170 (23.7) | 0.88 (0.63 - 1.23) | 0.45 |
|  |  |  |  |  |
| Block 3 ^b^ |  |  |  |  |
| - | - | - | - | - |
|  |  |  |  |  |
| Block 4 |  |  |  |  |
| A-G-C-G-A-A-T-G-G | 212 (30.5) | 243 (33.8) | 1 |  |
| A-G-C-A-T-A-T-G-A | 193 (27.7) | 220 (30.6) | 0.93 (0.69 - 1.25) | 0.62 |
| A-G-C-G-A-A-T-T-A | 109 (15.7) | 121 (16.9) | 0.97 (0.67 - 1.40) | 0.88 |
| A-G-T-G-T-A-T-G-A | 111 (15.9) | 87 (12.1) | 1.68 (1.13 - 2.48) | 0.010 |
| Rare ^c^ | 71 (10.2) | 47 (6.5) | 1.62 (1.00 - 2.62) | 0.050 |
