# Supplementary material for: A Non-Synonymous Single Nucleotide Polymorphism in the HJURP Gene Associated with Susceptibility to Hepatocellular Carcinoma among Chinese
Source: PLoS One. 2016 Feb 10;11(2):e0148618. doi: 10.1371/journal.pone.0148618 (PMC4749235; doi:10.1371/journal.pone.0148618)
Supplement: S7 Table — Healthy controls are non-HBV carriers negative for both hepatitis B surface antigen and antibody immunoglobulin G to hepatitis B core antigen. (DOCX) [file pone.0148618.s008.docx]

**S7 Table. The allele and genotype frequencies of** **rs3771333 in different populations.**

| Populations | Sample size |  | Allele, n (%) | |  | Genotype, n (%) | | |
| --- | --- | --- | --- | --- | --- | --- | --- | --- |
|  |  |  | A | C |  | AA | AC | CC |
| Chronic HBV carriers with HCC (Cases) | 448 |  | 758 (84.6) | 138 (15.4) |  | 317 (70.8) | 124 (27.7) | 7 (1.5) |
| Chronic HBV carriers without HCC (Controls) | 462 |  | 814 (89.1) | 100 (10.9) |  | 366 (80.1) | 82 (17.9) | 9 (2.0) |
| Healthy controls | 280 |  | 496 (88.6) | 52 (11.4) |  | 222 (79.3) | 52 (18.6) | 6 (2.1) |
